# Influence of orientation, size and shape of the region of interest in diffusion MRI along perivascular spaces index

**DOI:** 10.1007/s10334-025-01248-0

**Published:** 2025-04-11

**Authors:** Patricia Ulloa, Justus Christian Rudolf, Janina Kremer, Aileen Schmidt, Peter Schramm

**Affiliations:** 1 Department of Neuroradiology, University Hospital Schleswig-Holstein (UKSH), Ratzeburger Allee 160 | Haus A, 23538 Luebeck, Germany; 2 Department of Diagnostic and Interventional Radiology, University Hospital Bonn, Bonn, Germany

**Keywords:** Glymphatic system, Neurofluids, ALPS method, Interstitial fluid dynamics, DTI ALPS, DWI ALPS, Harmonization, ROI standardization

## Abstract

**Objective:**

Diffusion-tensor imaging (DTI) and diffusion-weighted imaging (DWI) along perivascular spaces (ALPS) index have been proposed as noninvasive techniques to indirectly evaluate the glymphatic system function. However, these techniques are sensitive to examination parameters, limiting inter-study comparability. The definition of the region of interest (ROI) has been identified as the primary weakness of the ALPS method. Therefore, we aimed to determine which ROI characteristics would best promote consistent ALPS index analysis.

**Methods:**

We examined 13 healthy volunteers using DTI and DWI to calculate the ALPS index, and compared and determined correlations among 11 different ROI configurations, and tested inter-method reliability.

**Results:**

We found significant differences between different ROI configurations in the ALPS index calculation. Considering ROI characteristics and inter-method reliability, a squared ROI is the most suitable. The ICC between ROI configurations showed good-to-excellent inter-method agreement (mean ICC = 0.83). We did not find significant inter-method differences.

**Conclusion:**

It is important to standardize the ROI characteristics for consistent ALPS index calculation.

**Supplementary Information:**

The online version contains supplementary material available at 10.1007/s10334-025-01248-0.

## Introduction

The glymphatic system [1,2] hypothesis aims to explain the waste clearance mechanism in the brain. Based on animal research [1,3], this hypothesis suggests that the cerebrospinal fluid (CSF) moves from the ventricles and the subarachnoid spaces through the perivascular spaces into the brain parenchyma. Then, CSF and interstitial fluid (ISF) are cleared along paravenous drainage pathways via aquaporin-4 water channels in the astrocyte’s feet, thereby eliminating toxic metabolites, such as amyloid-beta and tau-protein, out of the brain, mainly during sleep [1].

Glymphatic system function was initially evaluated noninvasively using the diffusion tensor image (DTI) along perivascular spaces (ALPS) index [4]. The ALPS method is based on the assumption that ISF movement in the deep white matter is present in the area near to the lateral ventricles and parallel to the paraventricular veins. Using a ratio between diagonal elements of the diffusion tensor in two regions of interest (ROI), it aims to remove the influence of the white matter fibers to evaluate the small diffusion component along the perivascular space [5]. The ALPS index has been widely studied in neurodegenerative diseases, such as Alzheimer’s [6,7] and Parkinson’s disease [8,9]. Furthermore, changes in the ALPS index have been reported between patients and healthy controls in cases of brain tumors [10,11], autism [12], and in non-neurological diseases, too. [13–16]

As for the DTI ALPS index, only diffusion along the x-, y-, and z-axes is needed to calculate the index; hence, there is no reason for not using DWI in three orthogonal gradient directions to obtain a DWI ALPS index when planning the imaging plane following the anterior commissure to posterior commissure (AC-PC) line [17]. DWI is easier and widely used in clinical routine and it has also been used to indirectly evaluate the glymphatic system in healthy volunteers [17] and for whole-brain radiotherapy [18], showing great potential in a fraction of the time needed for DTI.

However, there is significant heterogeneity across conventional DTI studies, indicating that standardized approaches are needed to homogenize clinical data for multicenter trials and meta-analysis [19]. Results of DTI-derived parameters (trace, fractional anisotropy) can be inconsistent and are difficult to compare when acquisition is not standardized [20].

Indeed, the lack of standardization for acquiring images and calculating the ALPS index could explain the current inter-study variability. Therefore, various studies have been conducted concerning suitability and standardization of measurements [17,21,22] to harmonize the ALPS index variations caused by scanner, site, and protocol differences. However, the definition of the ROI has been identified as the Achilles’ heel of the ALPS method [23], as it needs to be placed consistently. The ROI in the association area (corresponding to the longitudinal fasciculus) must avoid subcortical fibers and the projection area (corona radiata) should not be too close to the lateral ventricles: the presence of subcortical fiber or CSF would artificially increase the ALPS index.

In the literature, ROIs have been placed in the dominant hemisphere [24] and in both hemispheres [25]. Atlas-based placement [26,27], conversion to the standard brain registration [28], and automated ROI setup [29–32] have also been suggested. However, this is only possible when no major brain deformities or lesions are present. Furthermore, different ROI shapes and sizes have been used in previous studies: circular or spherical [4,25,32–35], squared or cubic or single voxel [13,36–38], crosses [39], and covering full projection and association areas over several slices [30]. Taoka et al. [17] compared ROIs of different sizes but not among different shapes (i.e., small sphere vs. large sphere, but not sphere vs. squared).

This raises the question of which kind of ROI to use when planning an ALPS study. In this work, our objectives were to assess how variations in the size and shape of the ROI affect the ALPS index calculation, investigate the differences and correlations in ALPS index, and compare the reliability and consistency of ALPS index values obtained from DTI and DWI.

## Experimental methods

### Subjects

This prospective study was approved by the ethical commission of the University of Luebeck, Germany (vote: 2023–416). Thirteen volunteers (8 women, 5 men; aged 22.8 ± 1.4 years) were informed about the project and possible risks and provided consent before the examination. The sample size was calculated based on the findings from Taoka et al. [17], who found significant inter-method differences with 7 volunteers. Under the assumptions of a large size effect (r = 0.82, estimated from Fig. 4a in Ref. [17]), significance level α = 0.05 and 80% power (1-β = 0.8), the required sample size was 11.65 participants. Adding 10% to account for non-normal distributed data, approximately 13 volunteers were considered sufficient to confirm significant inter-method differences.

### MRI data acquisition and analysis

The examination was performed on a 3T MRI scanner (MAGNETOM Vida, Siemens Healthineers, Germany), using a 20-channel head coil in neutral position (without tilt). The imaging parameters for DTI and DWI were: TE/TR = 92/4400 ms, FOV = 200 × 200 mm^2^, voxel size = 2 × 2 × 2 mm^3^, and *b*-values = 0 and 1000 s/mm^2^. The DTI measurement comprised 64 diffusion directions and DWI had three orthogonal directions in the readout, phase-encoding, and slice-encoding direction. Simultaneous multi-slice imaging with a total acceleration factor of 4 was used. The total durations of the DTI and DWI scans were 5:30 and 1:30 min, respectively.

The DTI images were analyzed using DSI Studio [40], version Apr. 23 2023 (http://dsi-studio.labsolver.org), and the DWI images were analyzed in ImageJ [41], version 1.54g (U.S. National institutes of health, Bethesda, Maryland, USA, https://imagej.nih.gov/ij/). In ImageJ, an image composite was generated with three channels: red, green, and blue for diffusivity along *x*-, *y*- and *z*-directions (in the readout, phase- and slice-selection coordinate system), as described by Taoka et al. [17].

### ALPS index calculation

The data were evaluated by a senior scientist (PU), placing the ROIs in a single slice per hemisphere, with ROIs aligned parallel within each hemisphere. Eleven ROIs were placed next to the lateral ventricle in the projection (superior corona radiata) and association (longitudinal fasciculus) areas according to a color-coded fractional anisotropy (FA) map by referring to susceptibility-weighted images to ensure the periventricular vessels were perpendicular to the lateral ventricle.

**Fig. 1:**
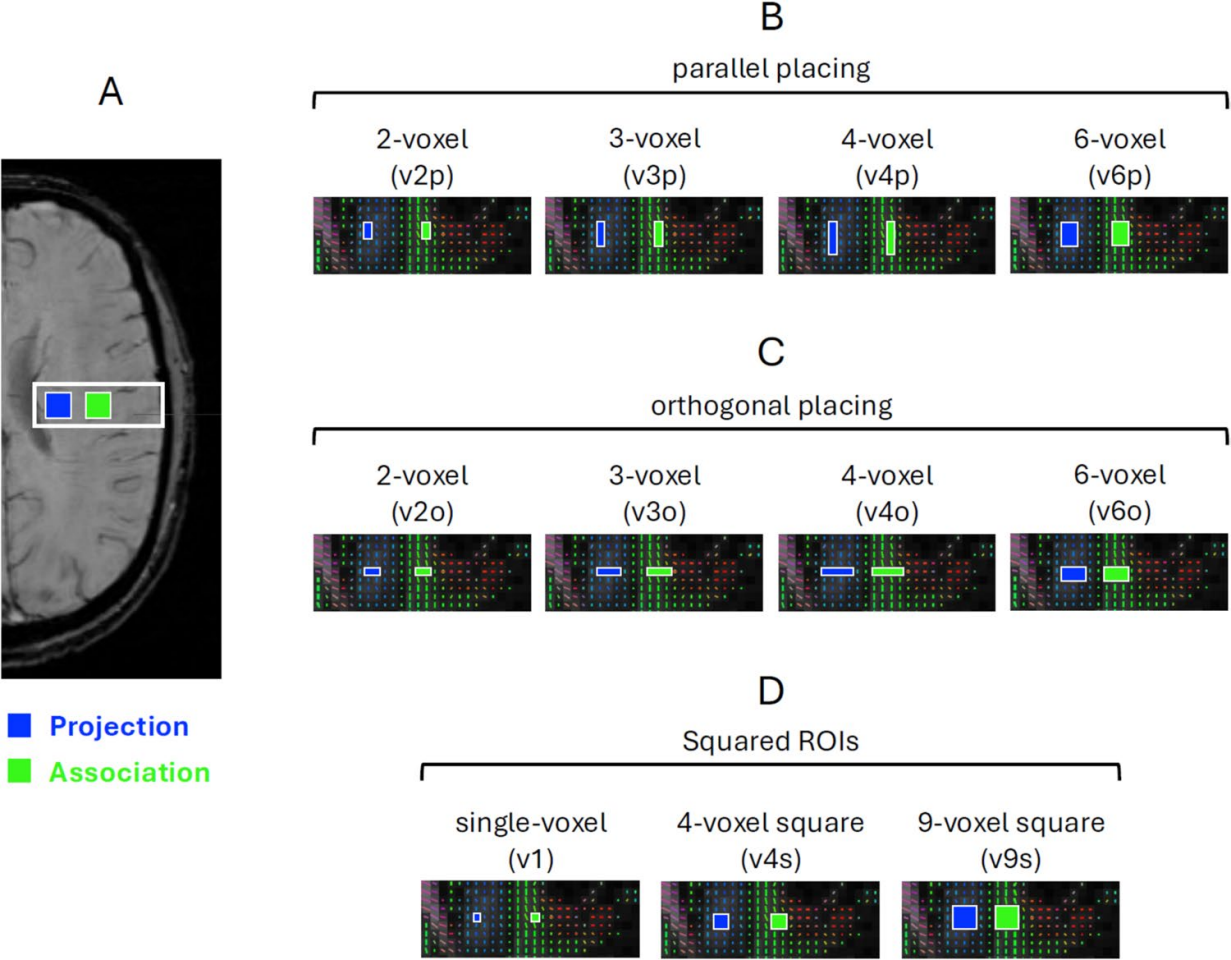
**A** Susceptibility-weighted image showing the position of the projection and association ROIs (in blue and green, respectively) using DSI Studio. **B**, **C** ROI configurations placed parallel and orthogonal to the lateral ventricle, respectively. **D** Squared ROI configurations. For simplicity, only the ROI placement on the left hemisphere is shown

The ROIs consisted of a single voxel (v1), 2, 3, 4, 6 and 9 voxels (with volumes of 8, 16, 24, 32, 48 and 72 mm^3^, respectively) and were placed parallel (v2p, v3p, v4p, v6p) and orthogonal (v2o, v3o, v4o, v6o) to the lateral ventricle, and as a 4-voxel and 9-voxel ROIs in a square configuration (v4s and v9s, respectively), as shown in Fig. 1. The 6-voxel ROIs, as they did not fit as a single row or column within the projection and association areas, were arranged in 3 × 2 or 2 × 3 voxel grids for parallel and orthogonal configurations, respectively. For DWI ALPS, we aimed to place the ROIs in the same slice (±1) as for DTI, with visualization of the corpus callosum, superior corona radiata, and longitudinal fasciculus based on the color-coded composite in ImageJ.

The projection ROIs were placed at least one voxel away from the lateral ventricle to avoid partial volume effect from the cerebrospinal fluid, which could artificially increase the ALPS index, and at least one voxel away from the association areas. Likewise, the association ROIs are at least one voxel away from the projection and subcortical regions.

From the tensor matrix, *x*-, *y*-, and *z*-axis diffusivities were obtained, and the DTI ALPS index was calculated as mean(DxProj,DxAssoc)/mean(DyProj,DzAssoc) [4], where Dx, Dy, and Dz correspond to the diagonal elements of the diffusion tensor. For the DWI ALPS index calculation, diffusivities along the readout (*x*–), phase- (*y*–), and slice-encoding (*z*–) directions were obtained directly from the scan and calculated using the same formula [17].

### Statistical analysis

Statistical analysis was performed using Matlab R2022a with a significance level of *α* = 0.05. Normality was determined using Lilliefors test. Differences were evaluated using the Wilcoxon signed-rank test.

We investigated interhemispheric differences in the DTI and DWI ALPS indices and between ROI configurations. In addition, we investigated the inter-method agreement (between DTI and DWI ALPS) using intraclass correlation coefficient (ICC) and Bland–Altman analysis. ICC estimates and their 95% confidence intervals (CI) for inter-method agreement were calculated using a two-way random-effects model to assess the agreement in a single rating. ICC values above 0.75 are considered good, values between 0.5 and 0.75 are considered moderate agreement, and values below 0.5 indicate poor reliability. Values above 0.9 are considered excellent agreement.

Bland–Altman analysis was performed to determine the average bias and the 95% limits of agreement between DTI and DWI. Limits of agreement (LOA) were calculated as bias ± 1.96 * SD, where SD is the standard deviation of the difference between paired samples.

## Results

### Descriptive statistics

**Fig. 2:**
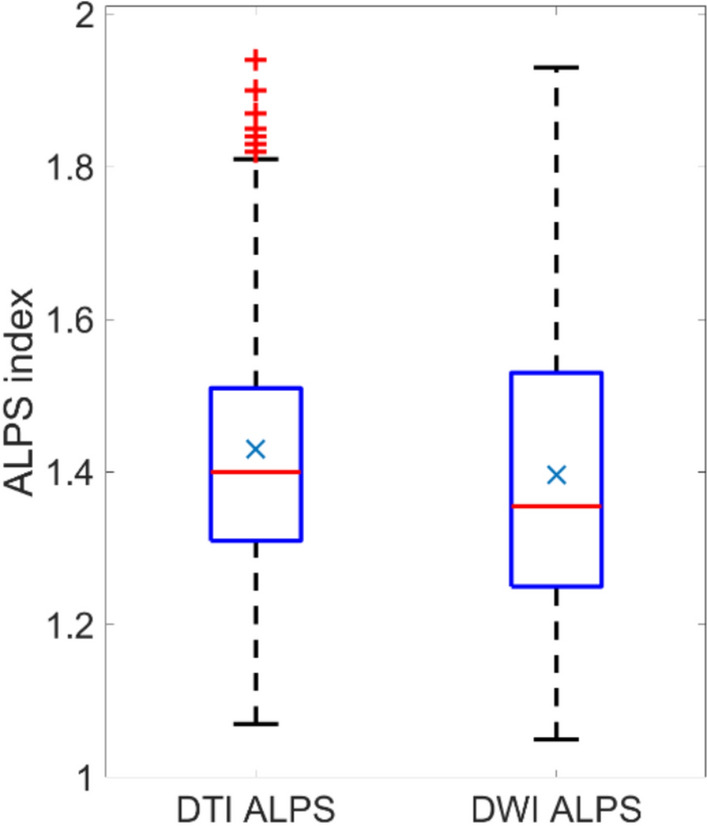
Box plot of ALPS indices results, summarizing both brain hemispheres, and all voxel configurations. Red line marks the median, x marks the mean, and red crosses are outliers

When considering the data from voxel configurations combined, but DTI and DWI separated, the datasets are not normally distributed (Lilliefors test, *p* < 0.01). Figure 2 summarizes the descriptive statistics for methods (DTI and DWI), considering both hemispheres and all voxel configurations combined. Result variability was observed between methods. The calculated DTI ALPS index tends to have a larger mean (1.43) and median (1.40) than the DWI ALPS index (1.40 and 1.36, for mean and median, respectively).

### Interhemispheric and inter-ROI configurations ALPS index comparison

In the DTI measurements, we did not find a significant difference between the right and left hemispheres (Wilcoxon signed-rank test, *p* > 0.1) (see Table 1). However, in DWI examinations, we found significant interhemispheric differences in ROIs v2o, v3o, and v3p (Wilcoxon signed-rank test, *p* < 0.04).

**Table 1:** Comparison between right and left hemispheres (Wilcoxon signed-rank test, p). Significant differences are marked with *. Separate analysis for DTI (right vs. left hemisphere), and DWI (right vs. left hemisphere) across different ROI configurations

	Right vs. left hemisphere (p)	
ROI configuration	DWI ALPS	DWI ALPS
v1	0.33	0.21
v2o	0.69	0.03*
v3o	0.39	0.03*
v4o	0.19	0.08
v6o	0.66	0.12
v2p	0.64	0.20
v3p	0.72	0.04*
v4p	1.00	0.20
v6p	0.37	0.32
v4s	0.44	0.07
v9s	0.41	0.22

Table S1 shows the p values from the Wilcoxon signed-rank test to evaluate ALPS index differences between different ROI configurations for DTI and DWI, for both hemispheres, separately. Only the voxel configurations that do not show significant differences across hemispheres and methods are shown. The ROI configurations consisting of a single voxel, v4s and v9s are “more stable”, making them more suitable for comparing methods.

### Inter-method and voxel configuration differences and correlation analysis

**Fig. 3:**
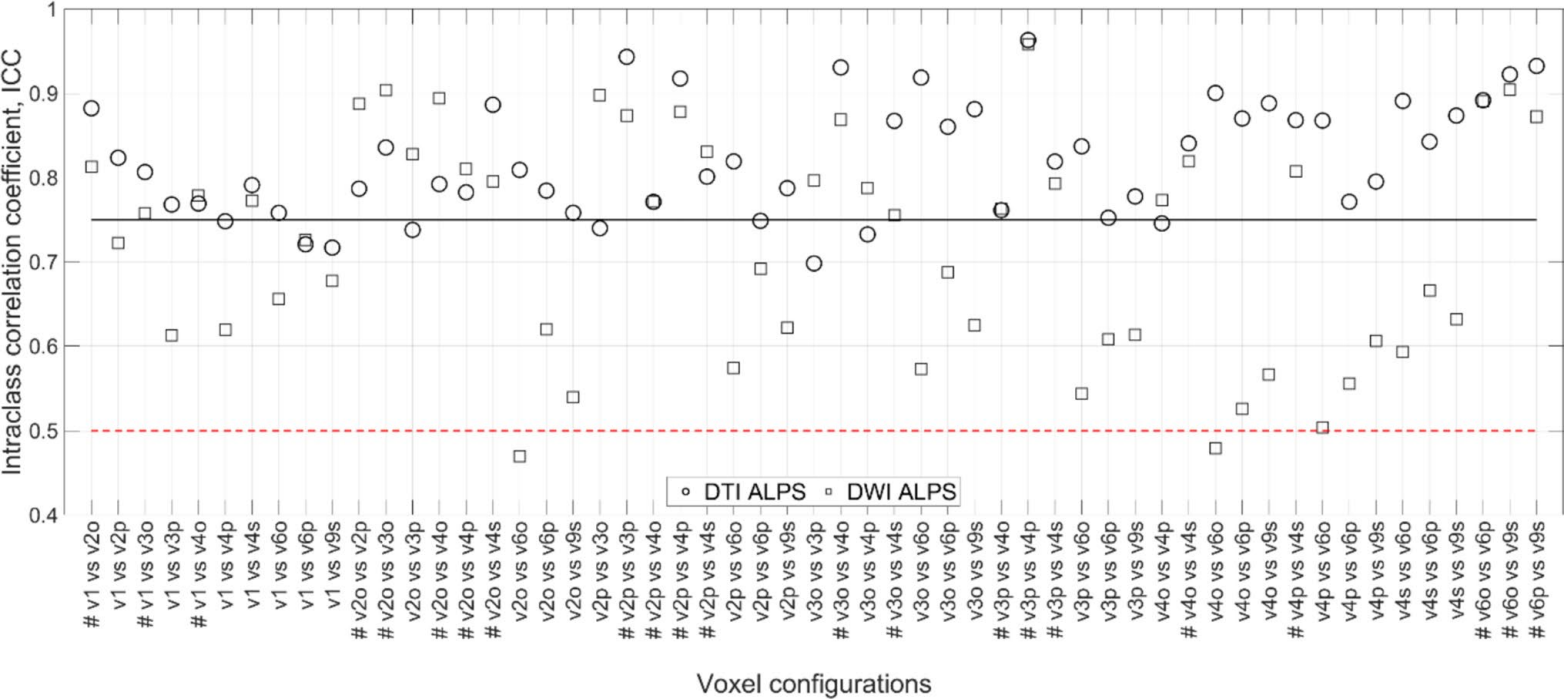
Plot showing the intraclass correlation coefficient (ICC) for different voxel configurations. The solid line represents ICC > 0.75, indicating good-to-excellent agreement. Voxel configurations marked with # indicate those that have ICC > 0.75 for all methods. The red dashed line represents ICC < 0.5, indicating poor agreement in two voxel configurations in the calculated DWI ALPS index. [Table S2 in suppl. material]

Figure 3 shows the ICC for all voxel configurations and methods. The ICC shows generally good agreement between methods and voxel configurations. ICC < 0.5 indicates poor agreement in two DWI ALPS calculations, corresponding to 3.64% of the measurements. ICC > 0.75 and ICC > 0.9 indicate good-to-excellent agreement in 96.36% of the inter-voxel configuration correlations. Twenty-two voxel configurations are marked with # to highlight the fact that this voxel configuration has a good-to-excellent agreement between methods with a mean ICC of 0.83. The 95% CI for the marked voxel configurations is shown in Table S2. As 95% CI has values larger than 1, some uncertainty in the ICC value is expected; however, in general, it is likely to be in the range indicating good-to-excellent reliability. The v4s voxel configuration shows higher reliability than other voxel configurations. However, among ROIs with larger volume, v9s is more reliable than v4s.

### Inter-method comparison and correlation analysis

Table 2 shows inter-method (DTI vs. DWI) comparison (Wilcoxon signed-rank test) for different ROI configurations. We reported no significant differences between DTI and DWI ALPS indices when comparing within the same ROI size and shape.

**Table 2:** Comparison between DTI and DWI ALPS index. Differences evaluated using Wilcoxon signed-rank test with α = 0.05. The evaluation using the average between right and left hemispheres. No significant inter-method differences were found

ROI configuration	DTI ALPS vs. DWI ALPS index (*p*)
v1	0.41
v2o	0.14
v3o	0.40
v4o	0.46
v6o	0.07
v2p	0.15
v3p	0.61
v4p	0.93
v6p	0.48
v4s	0.52
v9s	0.07

Nevertheless, there is poor inter-method agreement between the voxel configuration measurements (mean ICC = 0.42), as seen in Table 3. In addition, large F-test’s p values suggest that there might be inconsistencies in the measurements with the ROIs placed parallel to the lateral ventricle.

**Table 3:** Intraclass correlation coefficient (ICC), *p* value of the F-test (significant values are marked with a*) and 95% confidence interval (CI) for inter-method reliability

	ICC between DTI and DWI		
ROI configuration			95% CI
	ICC	F-test’s p	Lower, upper
v1	0.29	0.07	− 0.17, 0.75
v2o	0.13	0.26	− 0.38, 0.64
v3o	0.36	0.03*	− 0.07, 0.79
v4o	0.44	0.01*	0.02, 0.84
v6o	0.67	6.4e− 5*	0.36, 0.98
v2p	0.23	0.11	− 0.23, 0.71
v3p	0.31	0.06	− 0.14, 0.76
v4p	0.33	0.05	− 0.13, 0.77
v6p	0.54	1.72e− 3*	0.18, 0.91
v4s	0.53	0.003*	0.14, 0.90
v9s	0.80	3.32e− 7*	0.55, 1.04

However, the ICC between DTI and DWI is the largest (ICC = 0.80, *p* < 0.001) for v9s, suggesting excellent reliability between methods using 9 voxels in squared-shape ROI. Yet, the relatively wide 95% CI (0.55, 1.04) may indicate some uncertainty and variability across the sample.

### Bland–Altman analysis

**Fig. 4:**
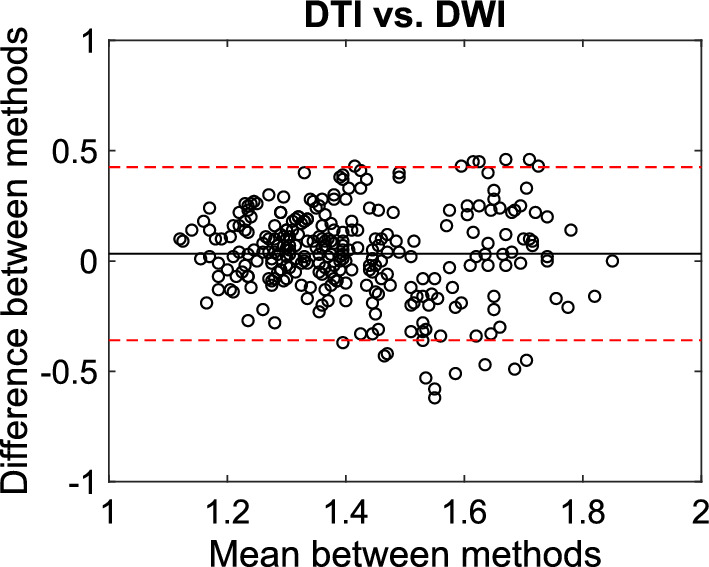
Bland–Altman plots illustrating the comparison of measurements across different methods (DTI vs. DWI)

Figure 4 shows the Bland–Altman plots showing DTI vs. DWI ALPS indices. The mean bias near zero (bias: 0.03) suggests minimal systematic differences between the two methods. The relatively narrow LOA (from − 0.36 to 0.43) indicates good agreement between DTI and DWI ALPS indices. This shows that the differences between the methods are generally consistent and within an acceptable range, with most differences falling within the expected limits.

## Discussion

We investigated the effect of ROI size and shape in the calculated ALPS index. In addition, we investigated the inter-method variability for ROIs of different sizes and shapes. In this work, we found no significant difference between left and right hemispheres in DTI and DWI in most of the ROI configurations in healthy volunteers. However, larger ROIs have the advantage of canceling out the spatial inhomogeneities in the brain parenchyma [17]. Therefore, v4s or v9s should be preferred, and asymmetric ROIs should be avoided for consistent ALPS index calculation.

Although the DTI and DWI ALPS indices may not differ significantly, they showed moderate reliability (ICC). This result contradicts the findings by Taoka et al. [17], where the comparison of the DTI and DWI methods showed significant differences but a high correlation. This contradiction might be explained by the fact that we did not average the two hemispheric indices, resulting in a higher ICC than a unilateral calculation. The interhemispheric ALPS index average could be appropriate for research performed on healthy volunteers. However, ALPS index studies in brain tumors [11], epilepsy [42], and stroke [8,28,43] have reported differences in the ALPS index between ipsi- and contralateral brain hemispheres. Therefore, when the area of the periventricular veins is intact, it is better to analyze the two hemispheres separately and report possible asymmetries.

The shape and relative orientation with respect to the lateral ventricle of the ROI has been shown to play a role in calculating the ALPS index for DTI and DWI. Particularly, ROIs placed orthogonal to the ventricle should not be compared to parallel, single-voxel, or squared ROIs. The partial inclusion of CSF, association, or subcortical fibers in the nearby ROIs might explain this finding, highlighting the need to carefully select ROI configurations and to standardize ALPS index calculation.

The squared ROIs are more reliable than other voxel configurations and show the highest inter-method reliability in the DTI ALPS index. However, the DWI ALPS index strongly depends on planning and positioning of the study subject in the scanner, which can result in ALPS index differences.

### Study limitations

We investigated only square or rectangular ROIs, as they provide consistent volume coverage, making it easy to compare ROIs of different sizes. This consistency is crucial for quantitative analysis. Squared or cubic ROIs have reduced edge effects, which is a problem with spherical or circular ROIs (leading to partial volume effects, where the voxel contains contributions from both inside and outside of the ROIs). However, we cannot discuss the impact of circular or spherical ROIs in the ALPS index calculation.

Our general recommendation is to use squared ROIs of 4 or 9 voxels. Larger ROIs have less placement variability, since there are fewer possible positions that do not overlap with adjacent areas. In contrast, smaller ROIs have greater variability in *x*- and *y*-directions, as they can be positioned in more ways without immediately overlapping other regions. However, a disadvantage of large ROIs (e.g., v9s) is that, in small brains, they can be difficult to place without including adjacent areas. In addition, we did not use any kind of interpolation, and unlike other studies, our voxel size was relatively big (2 × 2 × 2 mm^3^); therefore, it might be more appropriate to consider ROI volume rather than the number of voxels when interpreting the results.

In addition, our sample size calculation was based on the results of Taoka et al. [17]. However, unlike that publication, we did not find significant differences between DTI and DWI ALPS when comparing ROIs with the same configuration. This suggests that the size effect may be smaller than inferred from Ref. [17], and studies with a larger sample size might be required to draw more definitive conclusion regarding differences in the ALPS index calculation from DTI and DWI.

We used DSI Studio for DTI and ImageJ for DWI. Since DWI does not require complex analysis, the choice of software for ROI placement is not relevant, as long as it allows accurate ROI definition. However, in case of DTI, different software might use varying methods of calculating the diffusion tensor, leading to discrepancies in the resulting ALPS indices. Therefore, inter-software comparisons should also be conducted.

Furthermore, manual ROI placement is highly time consuming and demands significant human effort [21]. Therefore, manual ALPS index analysis becomes impractical in studies involving thousands of patients. To address this issue, automated calculation tools have been developed [39,44,45], demonstrating reproducible results [32,46], and should be preferred over manual ROI definition [26]. However, Carotenuto et al. [45] highlighted that while automatic ROI generation can reduce reader-dependent biases and improve efficiency, it might not be suitable for all cases. In multiple sclerosis patients, for example, due to white matter lesions, the ROIs should be placed manually to avoid the areas affected by demyelination. Therefore, consistent manual placement is still required in cases where white matter abnormalities prevent the use of automatic methods.

As a last point comes the limitations inherent to the ALPS method. As the ALPS index is calculated using small ROIs in deep white matter, the assumption that the ALPS index reflects the glymphatic system function of the whole brain is ambitious. In addition, accumulation of harmful metabolites occurs in cortical areas and cannot be assessed using the ALPS index. Thus, the meaning of the ALPS index has been called into question and should be confirmed using other methods [23].

## Conclusion

It is important to highlight that the DTI metrics primarily used, such as the measure of mean diffusivity in DTI protocols, are still not standardized and can lead to inconsistent results that are difficult to compare [47]. Yet, they have a high clinical value.

Our findings highlight the importance of standardizing ROI configurations to minimize variability, thereby improving the reliability of DTI and DWI measurements. Further research may be needed to identify other sources of variability and to develop strategies to enhance the consistency between these MR sequences.

Squared or cubic ROIs are generally more appropriate for MRI image analysis due to their alignment with the voxel grid, ease of implementation, reduced partial volume effects, and simplicity in statistical analysis. While there are specific cases where spherical or circular ROIs might be advantageous (such as tumor segmentation), the consistency and accuracy provided by squared or cubic ROIs often make them the preferred choice in quantitative imaging studies.

If researchers need to compare their findings with other published results, then it is better to use a v4s ROI. This ROI configuration showed good agreement with other ROI shapes and sizes. Using the v4s ROI as a standardized approach for ROI placement will bring the ALPS method one step closer to use in clinical routine. In addition, for inter-study comparison, it is also important to consider the total volume of the ROI, as we observed that large ROIs do not have excellent agreement with smaller ROIs.

Although recent developments have changed how the ALPS index is analyzed, indicating that it should not be directly related to glymphatic system function, it might represent a potential biomarker for diffusion in the perivascular space^23^. Indeed, various methods should be used in combination to study glymphatic system function.

## Supplementary Information

Below is the link to the electronic supplementary material.

Supplementary file1 (DOCX 18 kb)

## Data Availability

The data that support the findings could be available from the corresponding author upon reasonable request.
