# Supplementary material for: Influence of orientation, size and shape of the region of interest in diffusion MRI along perivascular spaces index
Source: MAGMA. 2025 Apr 11;38(5):761–9. doi: 10.1007/s10334-025-01248-0 (PMC12497668; doi:10.1007/s10334-025-01248-0)
Supplement: Supplementary file 1 — Supplementary file1 (DOCX 18 kb) [file 10334_2025_1248_MOESM1_ESM.docx]

**Influence of orientation size and shape of the region of interest in diffusion MRI along perivascular spaces index**

**Tables - Supplemental material**

**Table S1** Voxel configuration comparison using Wilcoxon signed-rank test for DTI and DWI ALPS indices and hemispheric differences. Only ROI configurations where no significant differences were found are shown (p > 0.05) between DTI and DWI measurements.

|  | DTI ALPS | | DWI ALPS | |
| --- | --- | --- | --- | --- |
| Voxel configuration | Right | Left | Right | Left |
| v1 vs. v2o | 0.88 | 0.35 | 0.62 | 0.38 |
| v1 vs. v6o | 0.56 | 0.09 | 0.77 | 0.12 |
| v1 vs. v2p | 0.41 | 1.00 | 0.72 | 0.62 |
| v1 vs. v3p | 0.85 | 0.90 | 0.53 | 0.90 |
| v1 vs. v4p | 0.68 | 0.82 | 0.39 | 0.66 |
| v1 vs. v6p | 0.50 | 0.33 | 0.74 | 0.93 |
| v1 vs. v4s | 0.44 | 0.39 | 0.42 | 0.83 |
| v1 vs. v9s | 0.69 | 0.22 | 0.91 | 0.74 |
| v2o vs. v6o | 0.57 | 0.42 | 0.57 | 0.44 |
| v2o vs. v2p | 0.22 | 0.40 | 0.43 | 0.92 |
| v2o vs. v3p | 0.54 | 0.35 | 0.57 | 0.14 |
| v2o vs. v6p | 0.70 | 0.66 | 0.86 | 0.39 |
| v2o vs. v4s | 0.20 | 0.83 | 0.66 | 0.16 |
| v2o vs. v9s | 0.62 | 0.72 | 0.48 | 0.80 |
| v3o vs. v4o | 0.90 | 0.53 | 0.48 | 0.96 |
| v6o vs. v4s | 0.11 | 0.10 | 0.35 | 0.08 |
| v2p vs. v3p | 0.80 | 0.68 | 0.11 | 0.11 |
| v2p vs. v6p | 0.43 | 0.46 | 0.56 | 0.49 |
| v2p vs. v4s | 0.77 | 0.28 | 0.16 | 0.37 |
| v2p vs. v9s | 0.56 | 0.21 | 0.88 | 0.70 |
| v3p vs. v4p | 0.57 | 0.79 | 0.39 | 0.08 |
| v3p vs. v6p | 0.78 | 0.30 | 0.51 | 0.80 |
| v3p vs. v4s | 0.65 | 0.33 | 1.00 | 0.95 |
| v3p vs. v9s | 0.91 | 0.20 | 0.20 | 0.58 |
| v4p vs. v6p | 0.72 | 0.30 | 0.44 | 0.65 |
| v4p vs. v4s | 0.75 | 0.31 | 0.80 | 0.51 |
| v4p vs. v9s | 0.67 | 0.25 | 0.08 | 0.28 |
| v6p vs. v4s | 0.72 | 0.55 | 0.77 | 0.62 |
| v6p vs. v9s | 0.47 | 0.96 | 0.57 | 0.51 |
| v4s vs. v9s | 0.63 | 0.66 | 0.37 | 0.46 |

**Table S2** Intraclass correlation coefficients (ICC) and 95% confidence interval (CI) for the voxel configurations that showed good-to-excellent reliability between voxel configurations and methods (F-test’s p-value < 0.01). Nonsignificant ICC are not shown (p > 0.05)

|  | DTI ALPS | | | DWI ALPS | | |
| --- | --- | --- | --- | --- | --- | --- |
|  |  | 95% CI | |  | 95% CI | |
| Voxel config. | ICC | lower | upper | ICC | lower | upper |
| v1 vs. v2o | 0.88 | 0.70 | 1.07 | 0.81 | 0.58 | 1.05 |
| v1 vs. v3o | 0.81 | 0.57 | 1.05 | 0.76 | 0.49 | 1.03 |
| v1 vs. v4o | 0.77 | 0.51 | 1.03 | 0.78 | 0.52 | 1.03 |
| v1 vs. v4s | 0.79 | 0.54 | 1.04 | 0.77 | 0.51 | 1.03 |
| v2o vs. v2p | 0.79 | 0.54 | 1.04 | 0.89 | 0.71 | 1.07 |
| v2o vs. v3o | 0.84 | 0.62 | 1.06 | 0.90 | 0.74 | 1.07 |
| v2o vs. v4o | 0.79 | 0.54 | 1.04 | 0.89 | 0.72 | 1.07 |
| v2o vs. v4p | 0.78 | 0.53 | 1.04 | 0.81 | 0.57 | 1.05 |
| v2o vs. v4s | 0.89 | 0.70 | 1.07 | 0.80 | 0.55 | 1.04 |
| v2p vs. v3o | 0.74 | 0.46 | 1.02 | 0.90 | 0.72 | 1.07 |
| v2p vs. v3p | 0.94 | 0.81 | 1.07 | 0.87 | 0.68 | 1.07 |
| v2p vs. v4o | 0.77 | 0.51 | 1.03 | 0.77 | 0.51 | 1.03 |
| v2p vs. v4p | 0.92 | 0.76 | 1.07 | 0.88 | 0.69 | 1.07 |
| v2p vs. v4s | 0.80 | 0.56 | 1.04 | 0.83 | 0.61 | 1.05 |
| v3o vs. v4o | 0.93 | 0.79 | 1.07 | 0.87 | 0.67 | 1.07 |
| v3o vs. v4s | 0.87 | 0.67 | 1.07 | 0.76 | 0.49 | 1.02 |
| v3p vs. v4o | 0.76 | 0.50 | 1.03 | 0.76 | 0.50 | 1.03 |
| v3p vs. v4p | 0.96 | 0.86 | 1.07 | 0.96 | 0.85 | 1.07 |
| v3p vs. v4s | 0.82 | 0.59 | 1.05 | 0.79 | 0.55 | 1.04 |
| v4o vs. v4p | 0.75 | 0.47 | 1.02 | 0.77 | 0.51 | 1.03 |
| v4o vs. v4s | 0.84 | 0.62 | 1.06 | 0.82 | 0.59 | 1.05 |
| v4p vs. v4s | 0.87 | 0.67 | 1.07 | 0.81 | 0.57 | 1.05 |
| v6o vs. v6p | 0.89 | 0.71 | 1.07 | 0.89 | 0.71 | 1.07 |
| v6o vs. v9s | 0.92 | 0.77 | 1.07 | 0.90 | 0.74 | 1.07 |
| v6o vs. v9s | 0.93 | 0.79 | 1.07 | 0.87 | 0.68 | 1.07 |
